# Identification of immune-related biomarkers in peripheral blood of schizophrenia using bioinformatic methods and machine learning algorithms

**DOI:** 10.3389/fncel.2023.1256184

**Published:** 2023-09-28

**Authors:** Xiaoli Zhu, Chuan-lan Wang, Jian-feng Yu, Jianjun Weng, Bing Han, Yanqing Liu, Xiaowei Tang, Bo Pan

**Affiliations:** ^1^The Key Laboratory of Syndrome Differentiation and Treatment of Gastric Cancer of the State Administration of Traditional Chinese Medicine, Yangzhou University Medical College, Yangzhou, China; ^2^Institute of Translational Medicine, Yangzhou University Medical College, Yangzhou, China; ^3^Tongzhou District Hospital of TCM, Nantong, China; ^4^Department of Psychiatry, Affiliated WuTaiShan Hospital of Yangzhou University Medical College, Yangzhou, China

**Keywords:** schizophrenia, peripheral immune-related biomarkers, CLIC3, WGCNA, CIBERSORT, LASSO, random forest, support vector machine

## Abstract

Schizophrenia is a group of severe neurodevelopmental disorders. Identification of peripheral diagnostic biomarkers is an effective approach to improving diagnosis of schizophrenia. In this study, four datasets of schizophrenia patients’ blood or serum samples were downloaded from the GEO database and merged and de-batched for the analyses of differentially expressed genes (DEGs) and weighted gene co-expression network analysis (WCGNA). The WGCNA analysis showed that the cyan module, among 9 modules, was significantly related to schizophrenia, which subsequently yielded 317 schizophrenia-related key genes by comparing with the DEGs. The enrichment analyses on these key genes indicated a strong correlation with immune-related processes. The CIBERSORT algorithm was adopted to analyze immune cell infiltration, which revealed differences in eosinophils, M0 macrophages, resting mast cells, and gamma delta T cells. Furthermore, by comparing with the immune genes obtained from online databases, 95 immune-related key genes for schizophrenia were screened out. Moreover, machine learning algorithms including Random Forest, LASSO, and SVM-RFE were used to further screen immune-related hub genes of schizophrenia. Finally, CLIC3 was found as an immune-related hub gene of schizophrenia by the three machine learning algorithms. A schizophrenia rat model was established to validate CLIC3 expression and found that CLIC3 levels were reduced in the model rat plasma and brains in a brain-regional dependent manner, but can be reversed by an antipsychotic drug risperidone. In conclusion, using various bioinformatic and biological methods, this study found an immune-related hub gene of schizophrenia – CLIC3 that might be a potential diagnostic biomarker and therapeutic target for schizophrenia.

## 1. Introduction

Schizophrenia is considered to be one of the most serious mental illnesses, affecting approximately 1% general population (McCutcheon et al., 2020). However, to date, the pathogenesis and molecular mechanism of schizophrenia still remain ambiguous. Early identification and diagnosis are likely to result in significant benefits for individuals with schizophrenia and the society (Gaebel and Zielasek, 2015). Therefore, identifying promising diagnostic biomarkers and therapeutic targets of schizophrenia is one of the focuses in future schizophrenia research.

Although the occurrence and development of schizophrenia is mainly located at the central nervous system (CNS), gene expression and metabolism in the peripheral blood of schizophrenia patients may also be affected through a wide range of cytokines, neurotransmitters, or hormones; in addition, the strong heritability of schizophrenia indicates the possible presence of detectable genetic biomarkers in peripheral blood (Kurian et al., 2011). Therefore, identifying genetic biomarkers in peripheral blood become a highly viable option in improving the diagnosis of schizophrenia.

There is growing evidence suggesting that the pathogenesis of schizophrenia may be related to a range of dysfunctional immune processes in the CNS and peripheral tissues (Khandaker et al., 2015). It was reported that indirect signs of immune dysregulation were presented in the CNS at the early stages of schizophrenia (Gangadin et al., 2019); hence, in the post-mortem brain samples of schizophrenia patients, biomarkers of neuroinflammation were detected (Trepanier et al., 2016). In addition, levels of pro-inflammatory biomarkers, such as cytokines, were shown to be elevated in the cerebrospinal fluid and peripheral blood of schizophrenia patients (Muller, 2018). Furthermore, increased levels of inflammatory cytokines were revealed to be associated with monocytes and macrophages in various psychiatric disorders, including schizophrenia (Goldsmith et al., 2016). More importantly, those immune-related changes in the CNS may originate from peripheral blood (Muller, 2018). Thus, identification of immune-related biomarkers in peripheral blood is of great significance for the diagnosis of schizophrenia.

**Figure 1:**
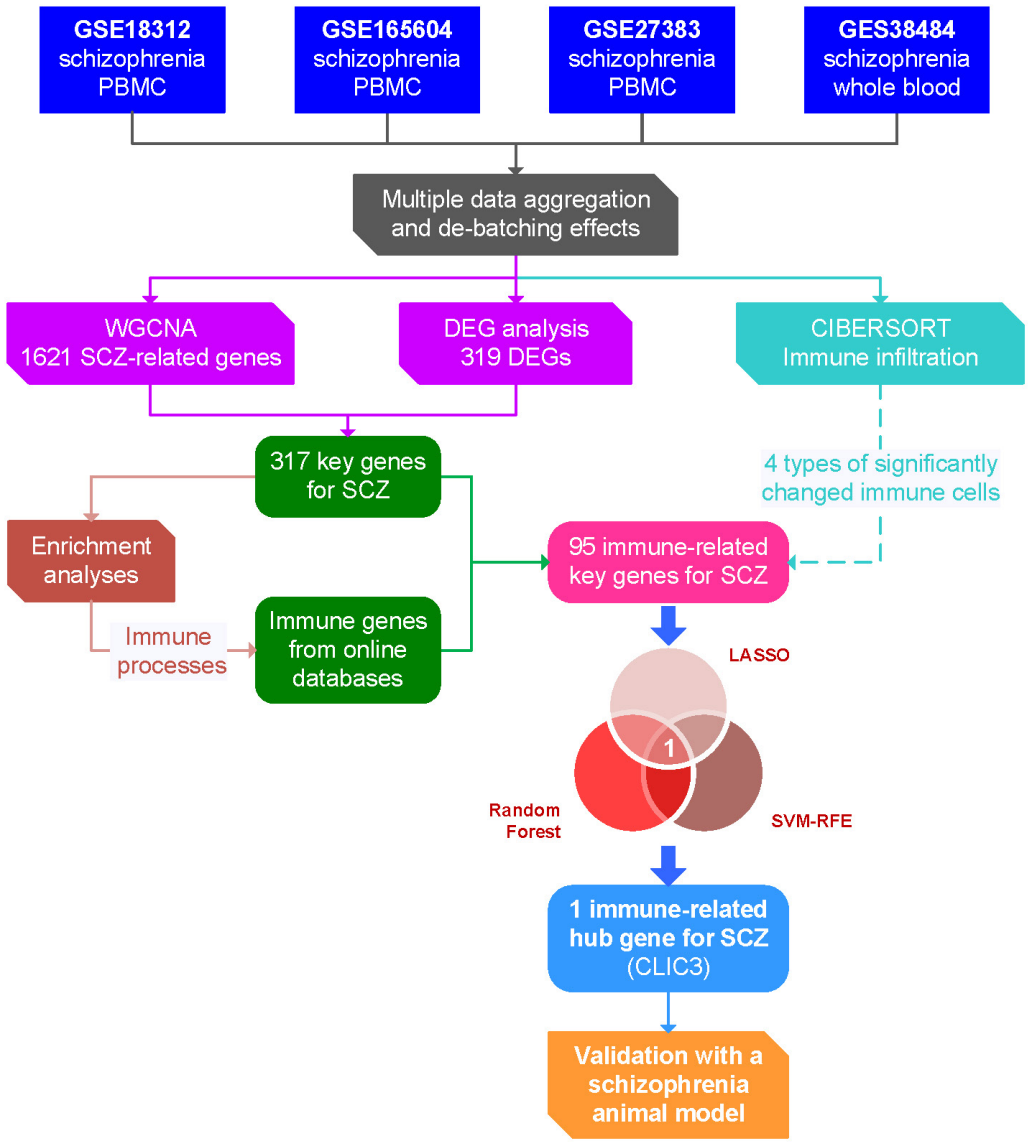
Flow chart of the present study. DEG, differentially expressed gene; LASSO, least absolute shrinkage and selection operator; PBMC, peripheral blood mononuclear cells; ROC, receiver operating characteristic; SCZ, schizophrenia; SVM-RFE, support vector machine – recursive feature elimination; WGCNA, weighted gene co-expression network analysis.

As shown in Figure 1, in the present study, four datasets of schizophrenia peripheral blood gene expression (GSE18312, GSE27383, GSE165604, and GSE38484) were selected from the Gene Expression Omnibus (GEO) database and combined into one dataset. A differentially-expressed gene (DEG) analysis and weighted gene co-expression network analysis (WGCNA) were performed with the combined dataset to screen key genes for schizophrenia, while an immune infiltration analysis was conducted to reveal significantly changed immune cell types using the CIBERSORT algorithm. Then, by comparing the key genes with the immune gene set from online databases, immune-related key genes for schizophrenia were obtained. Next, based on these immune-related key genes, three machine-learning algorithms were employed to identify hub gene (s) of schizophrenia, followed by *in vivo* validation of the hub gene (s). Overall, the present study might provide potential diagnostic biomarker (s) and therapeutic tactic (s) for schizophrenia.

## 2. Methods

### 2.1. Data processing

Four datasets of the mRNA expression profiles of the whole blood, blood lymphocytes, or peripheral blood mononuclear cells (PBMC) of schizophrenia patients and their corresponding healthy controls were downloaded from the NCBI’s GEO,^1^ including GSE18312 [GPL5175, Affymetrix Human Exon 1.0 ST Array], GSE165604 [GPL16791, Illumina HiSeq 2,500 (*Homo sapiens*)], GSE27383 [GPL570, Affymetrix Human Genome U133 Plus 2.0 Array], and GSE38484 [GPL6947, Illumina HumanHT-12 V3.0 expression beadchip]. The information of each dataset is demonstrated in Table 1.

**Table 1:** Information of the samples of the datasets.

Datasets	Samples	Controls	Schizophrenia	Method	Platform
GSE165604	Blood lymphocytes	18	19	RNA-seq	GPL16791
GSE27383	PBMC	29	43	Microarray	GPL570
GSE18312	PBMC	8	13	Microarray	GPL5175
GSE38484	Whole blood	96	106	Microarray	GPL6947

The four datasets contain a total of 309 samples, including 158 samples of schizophrenia patients and 151 samples of healthy controls (Table 1). The batch effects of these datasets were adjusted using the *COMBAT* function from the R package *inSilicoMerging* (Taminau et al., 2012). Then, a normalized merged expression matrix of the four datasets were generated and visualized by a box line plot. All these analyses were performed in the SangerBox platform.^2^

### 2.2. Weighted gene co-expression network analysis

WGCNA was performed to screen the key genes for schizophrenia (Langfelder and Horvath, 2008). Briefly, every gene of the merged expression matrix was calculated the MAD (Median Absolute Deviation) separately and eliminated the first 50% of genes with the smallest MAD. The *goodSamplesGenes* method from the R package *WGCNA* was used to remove outlier samples. The soft threshold power value was selected using the *pickSoftThreshold* function from the R package *WGCNA*, followed by building a scale-free co-expression network. Co-expression modules were constructed by dynamic tree cut and merge dynamic. The modules were composed of high-correlated genes and each module contained at least 30 genes. The correlation coefficient and *p* value of the module characteristic gene (ME) value with the clinical trait phenotype (Schizophrenia group and Control group) were calculated. Then, the relationships between gene significance (GS) and module membership (MM) were determined. The key schizophrenia-related module was determined by *p* value and correlation coefficient.

### 2.3. Identification of differentially-expressed genes

Differentially-expressed genes (DEGs) of the merged expression matrix were screened using the R package *limma* (Version 3.40.6) (Ritchie et al., 2015). The selection criteria of the DEGs included adjusted *p* value <0.05 and |log fold change (FC)| > 1.5. Heat map and volcano map of the DEGs were generated using the SangerBox platform.

### 2.4. Identification of hub genes and functional enrichment analysis

A Venn diagram (Bardou et al., 2014) was created using the SangerBox platform to analyze the intersection between the genes of the key WGCNA module and the DEGs to acquire schizophrenia-related key genes. Similarly, subsequent immune-related hub genes were also obtained using a Venn diagram. Gene Ontology (GO) and Kyoto Encyclopedia of Genes and Genomes (KEGG) enrichment analyses were then performed. The GO annotation of genes in the R package *org.Hs.eg.db* was used as the background, and the R package *clusterProfiler* was adopted to perform the enrichment analyses. The minimum gene was set to 5, the maximum gene was set to 5,000, and *p-*value <0.05 was considered statistically significant. Lastly, top 10 KEGG pathways (ranked by *p-*value) and top 10 GO terms (ranked by *p* value) were visualized using bubble charts, respectively.

### 2.5. Immune infiltration analysis

An immune cell infiltration analysis was performed using a machine learning method CIBERSORT.^3^ A total number of 1,000 iterations with LM22 labeling of genes were performed to quantify the corresponding proportions of 22 groups of immune cells. The composition of the 22 groups of immune cells was exhibited using multiple stacked histograms and the differential immune cells (*p* < 0.05) were selected and visualized using a violin plot.

### 2.6. Screening of immune-related hub genes

In the present study, the immune gene set was collected by combining and deduplicating three online datasets. The Pan-cancer immune genes were acquired according to a previous study (Charoentong et al., 2017). The innate immune system dataset was downloaded from PathCards of GeneCardsSuite^4^ after being retrieved by the keyword “immune.” The last gene set was obtained from the online database ImmPort.^5^

The machine-learning algorithms were performed in R. Hub genes were screened using three machine learning algorithms including Random Forest algorithm (Izmirlian, 2004), least absolute shrinkage and selection operator (LASSO) algorithm (Tibshirani, 1997), and support vector machines - recursive feature elimination (SVM-RFE) algorithm (Guyon et al., 2002). Random Forest algorithm was implemented using the R package *randomForest* (Version 4.7–1.1) (Breiman, 2001). The threshold for candidate hub genes was determined by the lowest point of the tenfold cross-validation error curve. The intersections of the genes with top-ranked mean decrease accuracy and those with top-ranked mean decrease Gini were considered as candidate genes. The R package *glmnet* (Version 4.1–7) was applied to implement the LASSO algorithm (Friedman et al., 2010; Simon et al., 2013). Candidate genes were selected by performing a tenfold cross-validation to adjust the optimal penalty parameter. Furthermore, the SVM-RFE approach was implemented using the R package *e1071* (Version 1.7–13).^6^ The parameters of the algorithm were set as follows: cost = 10, cachesize = 500, scale = F, type = “C-classificatio,” kernel = “linear.” The point of the lowest tenfold cross-validation error was selected as the threshold for candidate genes. Finally, the intersection gene(s) of the above three groups of candidate genes were selected as final hub gene(s).

The potential of the expression levels of the hub genes to differentiate schizophrenia patients from healthy controls was determined by a receiver operating characteristic (ROC) curve using GraphPad Prism 9.5.1. The area under curve (AUC) indicates the accuracy with which a particular hub gene can differentiate schizophrenia patients and controls. AUC values above 50% suggest that a hub gene can possibly differentiate between schizophrenia patients and controls and an AUC value of 100% indicates that it can perfectly differentiate between schizophrenia subjects and controls.

### 2.7. Animals, drug administration, and brain tissue dissection

Male Sprague–Dawley (SD) rats (aging 21 days and weighing 60 ± 5 g) were obtained from the Chang Cavens Laboratory Animals Co., Ltd. (Changzhou, Jiangsu, China). The rats were housed in a controlled environment (22 ± 1°C; light cycle from 07:00 AM to 07:00 PM) with food and water *ad libitum*. After 1-week acclimatization, the rats were randomly assigned into three groups (6 rats per group) and drug administration began. The experimental procedures of the present study were approved by the Animal Ethics Committee of Yangzhou University Medical College (Ethics No.: YXYLL-2020-53).

MK-801 is a non-competitive N-methyl-D-aspartic acid receptor (NMDAR) antagonist and commonly-used to establish NMDAR hypofunction models that mimic schizophrenia (Białoń and Wąsik, 2022). The drug administration was performed as described previously (Pan et al., 2022a,b, 2023a,b). Briefly, the rats except the controls received daily intraperitoneal injections of MK-801 (0.2 mg/kg/day, #M107, Sigma-Aldrich, St. Louis, MO, USA) between 10:00 AM and 10:30 AM for 2 weeks, while the control group were intraperitoneally injected saline (0.9%) for comparison. In our previous study, this modeling method induced schizophrenia-like abnormalities in male SD rats (Pan et al., 2022b). After the 2-week modeling, the risperidone group were orally administrated with risperidone (0.3 mg/kg/day, Xian Janssen Pharmaceutical Ltd., Xi’an, Shaanxi, China) three times a day (07:00 AM, 3:00 PM, and 11:00 PM) for another 2 weeks. Risperidone was orally delivered by mixing risperidone powder with a 0.2 g cookie dough pellet (containing corn flour, sugar, and milk powder). The other two groups were administrated with equivalent cookie dough pellets without risperidone. Two hours after the last administration, the rats were sacrificed and their blood and brains were collected and frozen for the subsequent biological experiments.

Prefrontal cortex (PFC), caudate putamen (CPu), nucleus accumbens (NAc), and hippocampus (HIP) are the brain regions that were reported to be closely associated with schizophrenia (Heckers, 2004; Bois et al., 2015; Mueller et al., 2015; Brady et al., 2019). In this study, the PFC, CPu, NAc, and HIP samples were dissected and collected using a cryostat (#CM1860, Leica Biosystems, Nussloch, Germany), as described previously (Pan et al., 2016, 2023a,b).

### 2.8. Enzyme-linked immunosorbent assay

CLIC3 levels in the rat plasma were detected using enzyme-linked immunosorbent assay (ELISA). Recombinant CLIC3 protein was used as standards. Blanks, standards, and plasma samples were loaded on a 96 well ELISA plate. Then, the plate was treated with an anti-CLIC3 antibody (1:500; #sc-390006, Santa Cruz, Dallas, TX, USA), a m-IgG2b BP-HRP secondary antibody (1:2000, #sc-542741, Santa Cruz), and a TMB (3, 3′,5,5’-Tetramethylbenzidine) solution (#P0210, Beyotime). Lastly, the plate was measured spectrophotometrically at 450 nm on a microplate reader. All standards and samples were run in duplicate to ensure consistency.

### 2.9. Western blots

Western blots were performed as described in our previous studies (Pan et al., 2016, 2022a,b, 2023a; Pan and Deng, 2019). Briefly, the brain samples were homogenized in a NP-40 homogenizing buffer (#P0013F, Beyotime, Shanghai, China) containing Protease Inhibitor Cocktail (#P8340, Sigma-Aldrich). Then, the homogenized samples were denatured under 95°C to prepare loading samples. The total protein concentration in each sample was detected using a Bradford protein assay kit (#P0006, Beyotime). Loading samples containing 15 μg of total protein were loaded into a 12% SDS-PAGE gel and then transferred to a polyvinylidene difluoride (PVDF) membrane. The PVDF membranes were blocked by 5% skim milk for 2 h at room temperature and incubated with a primary antibody overnight at 4°C. The immunoreactive signals were examined by the ChemiDoc XRS+ System (Bio-Rad, Hercules, CA, USA) and quantified by ImageLab Software (Bio-Rad, Version 6.1). The data were normalized with their corresponding GAPDH levels and then transferred by taking the value of the control group as 100%. The images of the uncut membranes used in this study are shown in the Supplementary Figure S1.

An anti-CLIC3 primary antibody (1:1000; #15971-1-AP, Proteinech) was purchased and used to detect CLIC3 protein expression in the four rat brain regions. A mouse anti-GAPDH monoclonal antibody (1:50000; #60004-1-lg, Proteintech) was used to determine GAPDH levels. A secondary HRP-linked anti-mouse IgG antibody (1:2000; #7076, Cell Signaling) and HRP-linked anti-rabbit IgG antibody (1:2000; #7074, Cell Signaling) were used to generate immunoreactive signals.

### 2.10. Statistical analysis

All bioinformatic analyses in the present study were performed either in R or in the SangerBox platform, as described in the previous sections. The data of the *in vivo* experiments were analyzed and visualized using Prism GraphPad (Version 9.5.1) (GraphPad Software, San Diego, CA, USA). One-way analysis of variance (one-way ANOVA) analysis was performed, followed by performing post-hoc Dunnett’s *t-*tests to compare each group with their corresponding model (MK-801) groups. Statistical significance was accepted when *p*-value was less than 0.05. All *in vivo* experiments were performed in triplicate to ensure consistency.

## 3. Results

### 3.1. Multiple data aggregation and de-batch effects

**Figure 2:**
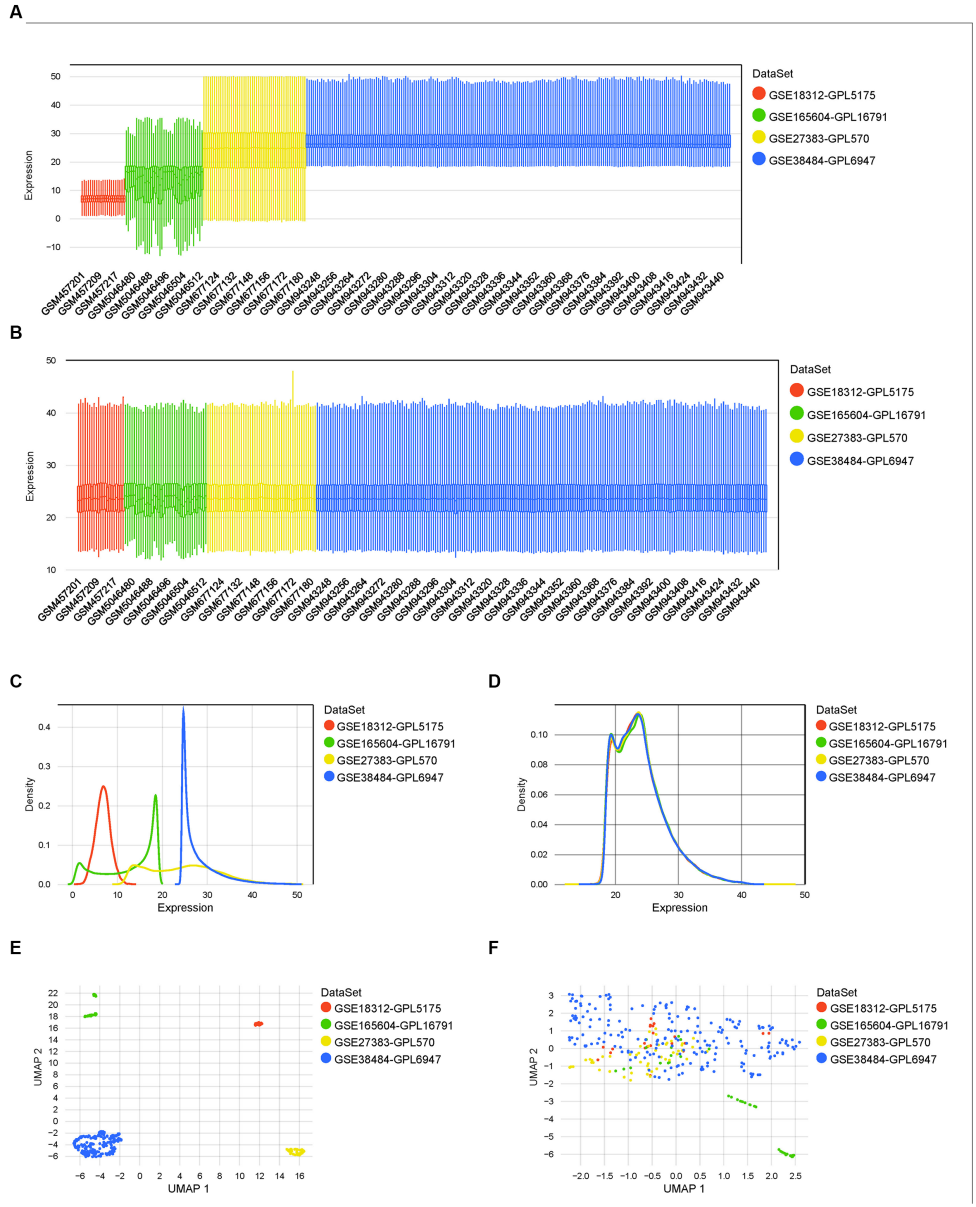
Normalization, merging, and de-batching of the four datasets. **(A,B)** Boxplots of the four datasets before and after removing the batch effect. **(C,D)** Density maps of the four datasets before and after removing the batch effect. **(E,F)** UMAP maps of the four datasets before and after removing the batch effect.

Four datasets of the mRNA expression of the whole blood, blood lymphocytes, or PBMC of schizophrenia patients and matched controls were included in the present study. The boxplot (Figure 2A), density plot (Figure 2C), and UMAP plot (Figure 2E) of the four datasets exhibited that the sample distribution of each dataset was quite different, indicating the existence of a batch effect. After the empirical Bayesian method *COMBAT* was employed to remove the batch effect, the data distribution between the datasets tended to be consistent. Specifically, the median of each dataset was basically on a line (Figure 2B); the mean and variance of the four datasets became similar (Figure 2D); and, the samples are clustered and intertwined (Figure 2F), which together indicated a satisfying de-batching effect.

### 3.2. Screening of schizophrenia-related genes using WGCNA

**Figure 3:**
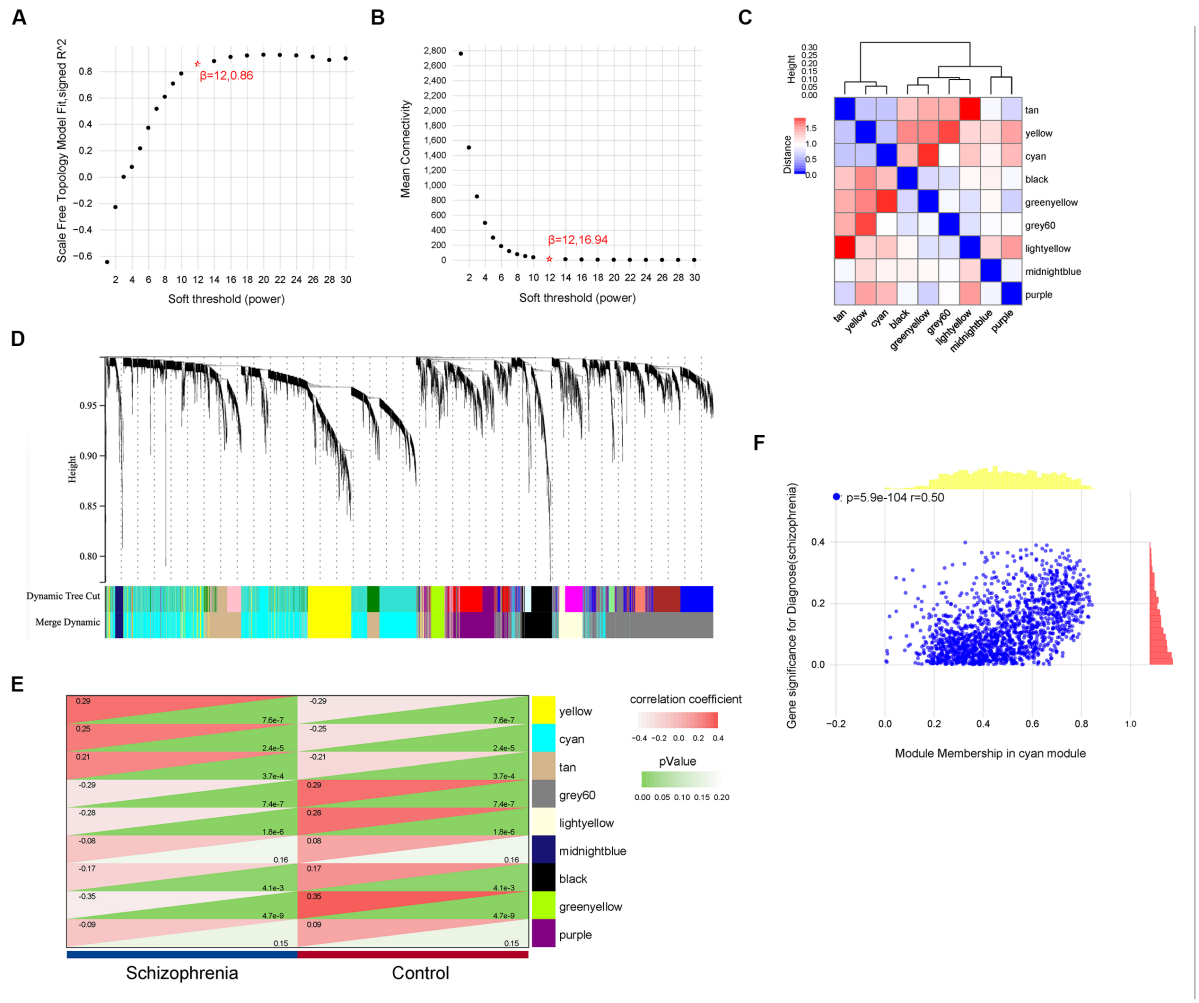
Weighted gene co-expression network analysis. **(A,B)** The values of soft-threshold power based on scale independence and mean connectivity of the weighted gene co-expression network analysis (WGCNA). **(C)** Nine co-expression modules of WGCNA. **(D)** Cluster dendrogram of genes of WGCNA. Each color represented a module, and the gray module included the genes that could not be classified into any module. **(E)** Heatmap of the eigengene network representing the relationships among the modules and the clinical trait status Heatmap of correlations between module characteristic genes (MEs) and phenotype of clinical traits (type of disease). Red represented correlation and green represented *p-*value. **(F)** Correlation of gene significance (GS) and module membership (MM) of the cyan module.

For a more precise follow-up analysis, 10,543 genes from a total of 309 samples were obtained by combining the four datasets (Supplementary Table S1). The top 5,272 most distinct genes were selected to build a co-expression network using WGCNA (Figure 3). The soft threshold power was set to 12, by which the scale independence reached 0.86 and the average connection value was 16.94 (Figures 3A,B). Additionally, the module sensitivity was set to 1.0, the module pooling threshold was set to 0.5, and the minimum module gene number set to 30. Finally, 9 co-expression modules were identified (Figure 3C). There were 277 genes in the black module, 1,621 genes in the cyan module, 132 genes in the greenyellow module, 215 genes in the lightyellow module, 78 genes in the midnightblue module, 489 genes in the purple module, 720 genes in the tan module, 467 genes in the yellow module, and 1,273 genes in the grey60 module (gene expression and sample matrix information in Supplementary Table S2). The grey module, consisting of non-co-expressed genes, was considered as an invalid module and excluded from the following analysis (Figure 3D). Depending on the ME values of the obtained modules, the correlation between these modules and the clinical trait (schizophrenia vs. control) was performed. The cyan module, possessing a high correlation with schizophrenia (*r* = 0.25, *p* = 2.4e-5) and the largest number of genes among these modules, was selected as a key module for the subsequent analysis (Figure 3E). The relationship between MM and GS was evaluated in the key module with a correlation coefficient of 0.50 and a *p*-value of 5.9e-104 (Figure 3F).

### 3.3. DEG analysis and identification of schizophrenia-related key genes

**Figure 4:**
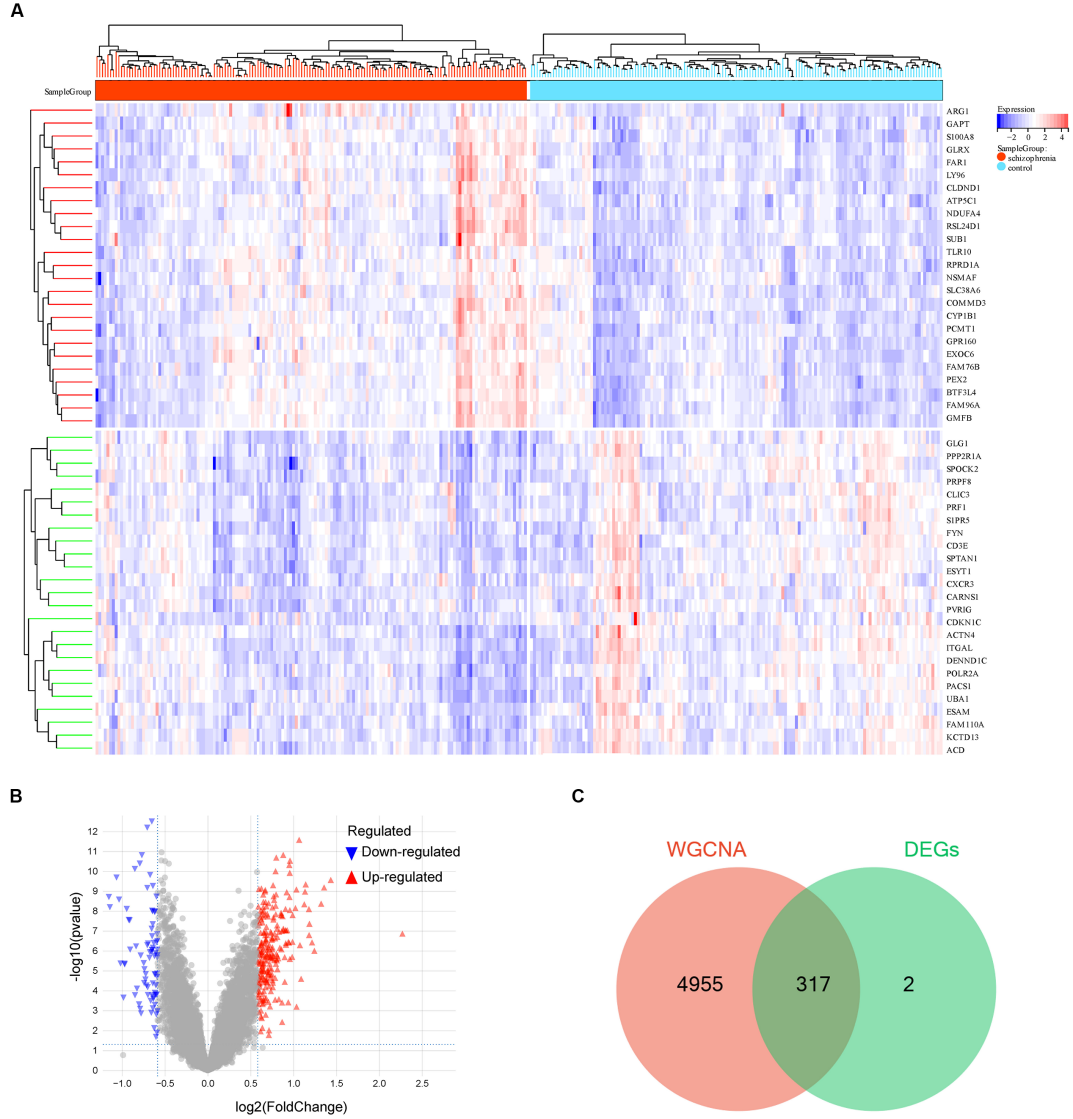
Differentially expressed gene analysis. **(A)** Hot map of the differentially expressed genes (DEGs). **(B)** Volcano map of the DEGs. **(C)** Venn diagram of the genes screened by weighted gene co-expression network analysis (WGCNA) and DEGs. −, *p* > 0.05; *p < 0.05; **p < 0.01; ***p < 0.001; ****p < 0.0001.

The DEG analysis for the 10,543 genes were performed using the R package *limma*. The top 25 up-regulated and down-regulated DEGs (ranked by logFC) are shown in Figure 4A. Totally, 319 DEGs were identified, including 237 up-regulated and 82 down-regulated DEGs (Figure 4B and Supplementary Table S3). Furthermore, we used a Venn diagram to screen and visualize the schizophrenia-related key gene by intersecting the DEGs with the genes of the cyan module of WGCNA. Finally, 317 schizophrenia-related key genes were obtained (Figure 4C and Supplementary Table S4).

### 3.4. Functional enrichment analyses

**Figure 5:**
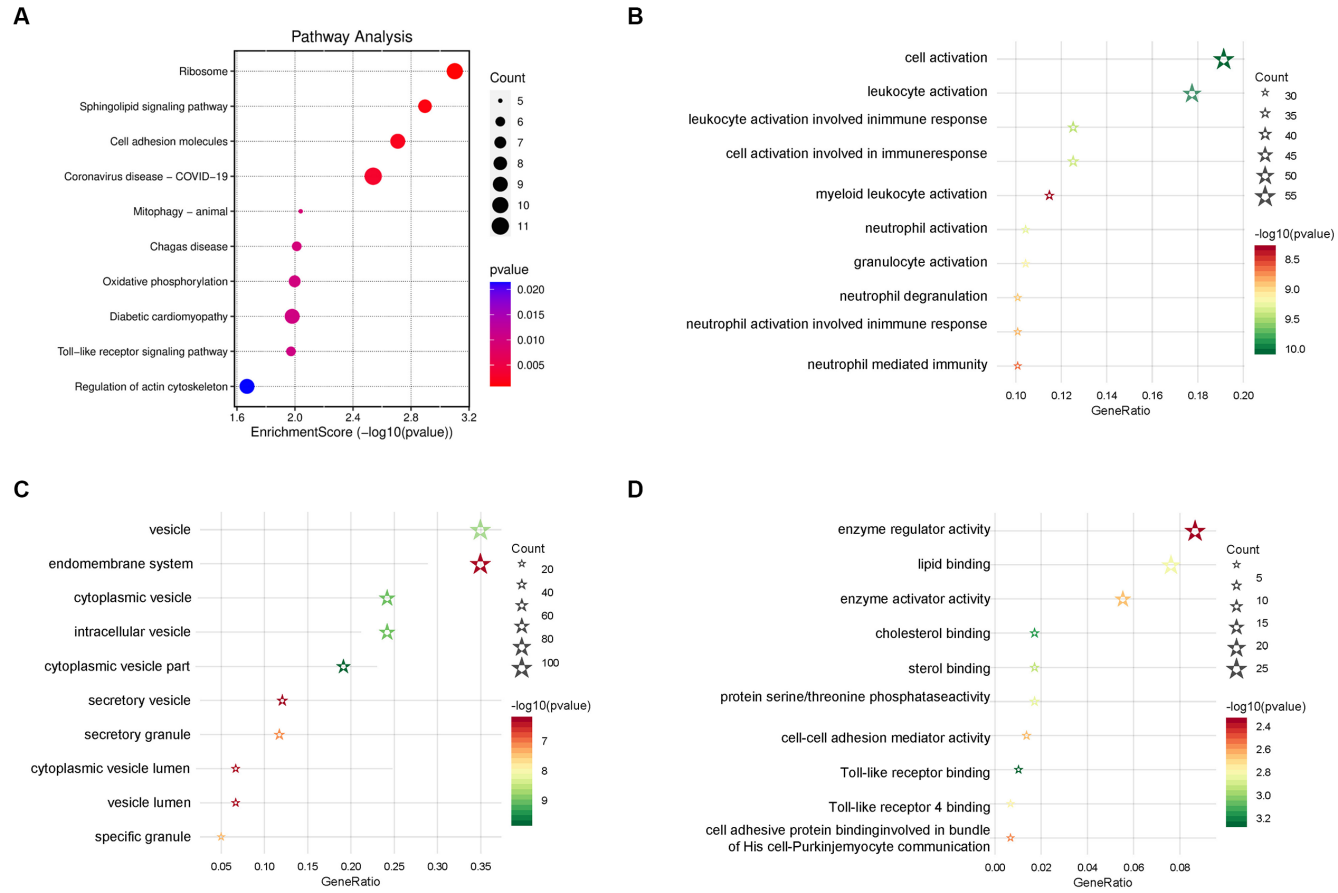
Functional enrichment analyses. **(A)** Signaling pathways of the Kyoto Encyclopedia of Genes and Genomes (KEGG) enrichment analysis. **(B)** Biological processes of the Gene Ontology (GO) enrichment analysis. **(C)** Cellular components of the GO enrichment analysis. **(D)** Molecular function of the GO enrichment analysis (the color of the bubble represents the *p-*value, and the size of the bubble represents the number of genes).

In order to clarify the biological function of the schizophrenia-related key genes obtained in the previous step, KEGG and GO enrichment analyses on these genes were performed. The KEGG pathway enrichment analysis showed several schizophrenia-related signaling pathways, including ‘cell adhesion molecules (CAMs),’ ‘toll-like receptor signaling pathway,’ ‘platelet activation,’ ‘oxidative phosphorylation,’ and ‘apoptosis’ (Figure 5A and Supplementary Table S5). The GO enrichment analysis revealed that these genes were largely enriched in the biological functions related to immune activities, such as ‘cell–cell adhesion mediator activity,’ ‘leukocyte activation involved in immune response,’ ‘cell activation involved in immune response,’ ‘neutrophil activation involved in immune response,’ ‘neutrophil mediated immunity,’ ‘leukocyte activation,’ ‘neutrophil activation,’ and ‘granulocyte activation’ (Figures 5B–D and Supplementary Table S6). These enrichment analyses together suggest that schizophrenia may be related to cell cycle, vesicle trafficking, apoptosis, and particularly immune-related signaling pathways. Besides, the results also showed that the DEGs are also involved in some other molecular pathways that are associated with schizophrenia. For example, PPP2R1A, NSMAF, S1PR5, FYN, PTEN, PPP2R5A, GNA13, and PLCB2 are involved in the ‘Sphingolipid signaling pathway’; RHOT1, BNIP3L, TAX1BP1, MFN1, and BCL2L1 are associated with ‘Mitophagy’; NDUFA4, NDUFB3, COX7A2, PPA2, UQCRQ, ATP6V1G1, and COX7B are related to the process of ‘Oxidative phosphorylation’; and, SPTAN1, PRF1, CTSW, CTSK, GZMB, and BCL2L1 are linked to ‘Apoptosis.’ Interestingly, some DEGs, such as RSL24D1, RPS15A, RPLP0, IRF3, RPL31, RPL23, RPL15, RPS27L, RPS4Y1, TLR8, and RPS24, are found to be associated with ‘Coronavirus disease - COVID-19’. A previous study found that severe COVID-19 could increase risk for schizophrenia and suggested that schizophrenia should be assessed as one of the post-COVID-19 sequelae (Baranova et al., 2022). Therefore, the current findings strengthened the genetic link between schizophrenia and COVID-19 and revealed possible genes that are involved in.

### 3.5. Immune infiltration analyses and screening of immune-related key genes of schizophrenia

**Figure 6:**
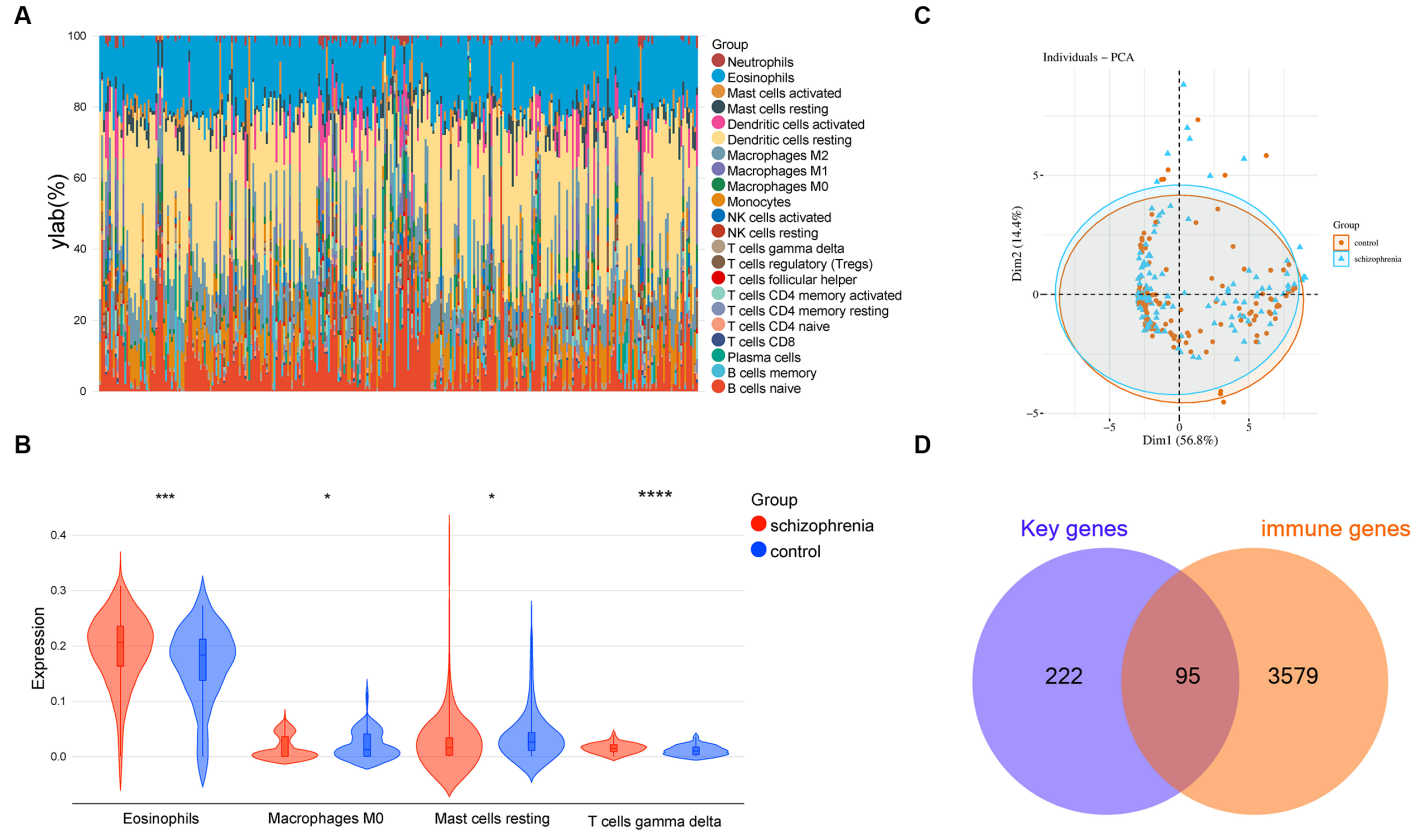
Immune cell infiltration analysis. **(A)** Relative percentage of 22 groups of immune cells in each sample. **(B)** Differences in immune infiltration between schizophrenia and control samples, including 4 significantly different immune cell groups: eosinophils, M0 macrophages, resting mast cells, and gamma delta T cell. **(C)** Principal component analysis for the immune cell infiltration between schizophrenia subjects and healthy controls. **(D)** Venn diagram indicating 95 immune-related key genes for schizophrenia. −, *p* > 0.05; *p < 0.05; **p < 0.01; ***p < 0.001; ****p < 0.0001.

The enrichment analyses indicated that immune-related biological functions are particularly related to schizophrenia, therefore, we further used a machine learning algorithm CIBERSORT to analyze the immunological features of the datasets. The multi-group stacked histogram shows the distribution and infiltration abundance of 22 groups of immune cells in the 309 samples (Figure 6A). Among them, eosinophils, M0 macrophages, mast cells testing, and gamma delta T cells were revealed to be significantly different between the schizophrenia subjects and heathy controls (Figure 6B). A principal component analysis (PCA) indicated that the immune cell infiltration of the schizophrenia group is significantly different from that of the controls (Figure 6C). Moreover, by intersecting the immune genes from online databases with the key schizophrenia-related genes obtained in the previous step, a total of 95 immune-related key genes for schizophrenia were obtained (Figure 6D and Supplementary Table S7).

### 3.6. Screening and validation of immune-related hub gene(s) for schizophrenia

**Figure 7:**
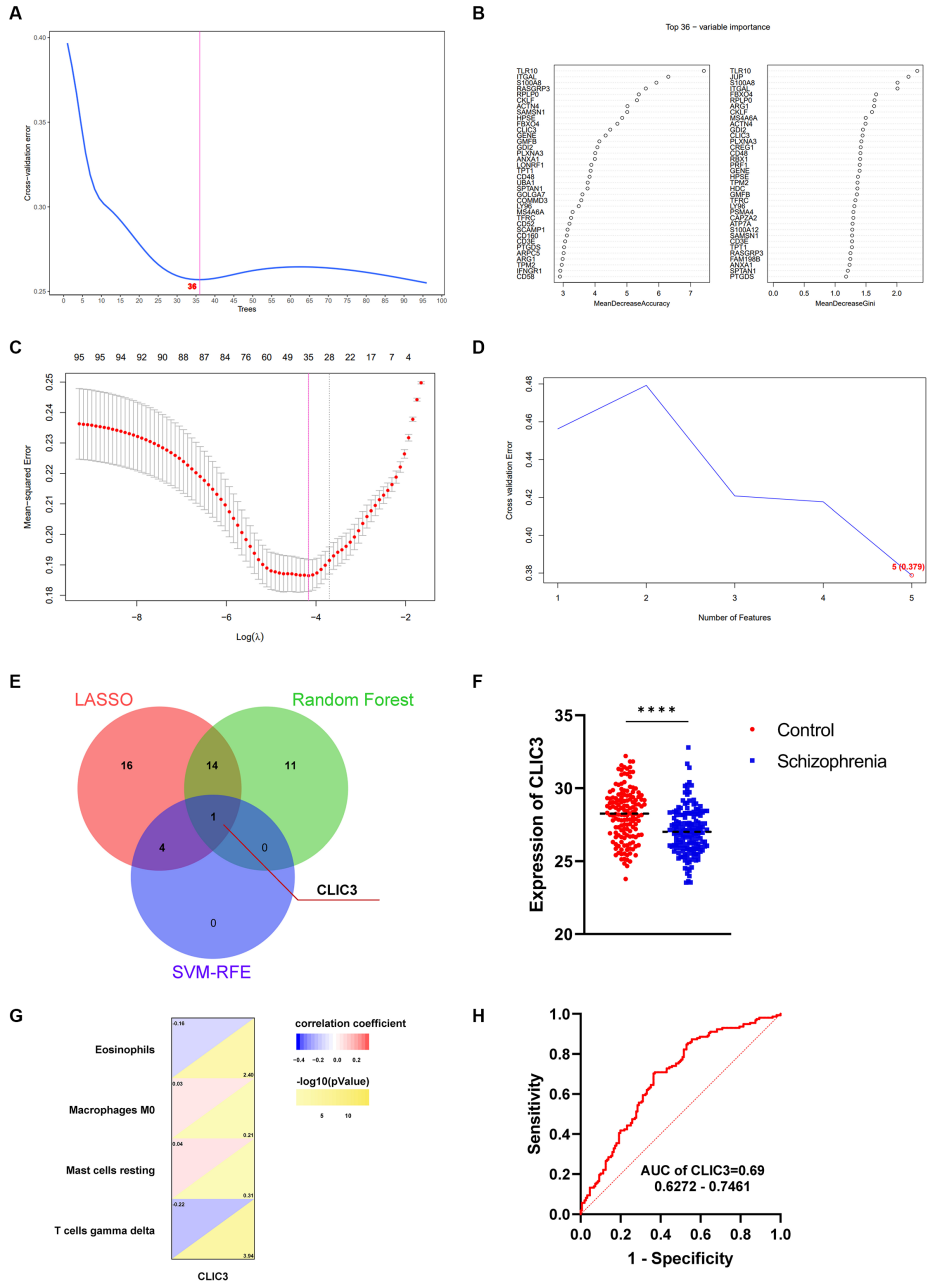
Identification of immune-related hub genes for schizophrenia. **(A)** The Random Forest analysis indicates 36 candidate hub genes. **(B)** The 36 candidate hub genes generated by the Random Forest algorithm are ranked by ‘MeanDecreaseAccuracy’ (left) and ‘MeanDecreaseGini’ (right), respectively. **(C)** The least absolute shrinkage and selection operator (LASSO) analysis indicates 35 candidate hub genes. **(D)** The support vector machines - recursive feature elimination (SVM-RFE) analysis indicates 5 candidate hub genes. **(E)** A Venn diagram indicating one hub gene of schizophrenia by intersecting the three groups of candidate genes revealed by the three machine-learning algorithms, respectively. **(F)** The mRNA expression of CLIC3 between the schizophrenia patients and healthy controls (****p < 0.0001). **(G)** The relationship between CLIC3 and immune cells. **(H)** The ROC curve of CLIC3 to assess the accuracy of CLIC3 to potentially differentiate between the schizophrenia patients and healthy controls.

Three Machine learning algorithms including Random Forest, LASSO, and SVM-RFE were further employed to screen hub genes using the 96 immune-related signature genes of schizophrenia. First, according to the lowest point of the tenfold cross-validation error curve of the Random Forest algorithm, the number of candidate genes was 36 (Figure 7A). In addition, by intersecting the genes with top-ranked mean decrease accuracy and those with top-ranked mean decrease Gini (Figure 7B), a total of 27 genes were acquired as candidate hub genes (Table 2). Second, the LASSO analysis identified 35 candidate hub genes as indicated by the lowest point of the tenfold cross-validation error curve (Figure 7C and Table 2). Third, the cross-validation error curve of the SVM-RFE algorithm revealed 5 candidate hub genes (Figure 7D and Table 2). Of these 3 candidate groups, 1 intersection gene was acquired as the immune-related hub genes of schizophrenia, which was CLIC3 (Figure 7E). Back to the mRNA expression data, the mRNA expression of CLIC3 was significantly decreased in the schizophrenia samples compared to the healthy controls (Figure 7F).

**Table 2:** Candidate hub genes for schizophrenia screened out by the random forest, LASSO, and SVM-RFE algorithms.

Random forest	LASSO	SVM-RFE
TLR10, S100A8, ITGAL, FBXO4, RPLP0, CKLF, MS4A6A, ACTN4, GDI2, CLIC3, ARG1, PLXNA3, CD48, GENE, HPSE, TPM2, GMFB, TFRC, LY96, SAMSN1, CD3E, TPT1, RASGRP3, ANXA1, SPTAN1, PTGDS	S100A8, SPTAN1, TLR10, ARG1, CLIC3, SAMSN1, CD3E, RPLP0, CREG1, TFRC, CXCR3, CKLF, HPSE, ABCA1, GOLGA7, CD48, TAX1BP1, ATP7A, JUP, ATP8B4, RASGRP3, PLXNA3, ARPC5, CTSK, OGT, CD160, PTGS2, S100A12, HDC, LYZ, GCA, ELANE, PROK2, IFITM3	GCA, HDC, CLIC3, ARPC5, TAX1BP1

LASSO, least absolute shrinkage and selection operator; SVM-RFE, support vector machines - recursive feature elimination.

Correlation analyses were performed to reveal the relationship between CLIC3 and the 4 significantly different immune cells discovered in the previous step (Figure 7G). The analysis revealed that CLIC3 was negatively correlated with eosinophil and gamma delta T cells and positively correlated with M0 macrophages and resting mast cells.

ROC curves of the immune-associated hub genes for schizophrenia were drawn to show the potential of these hub genes to differentiate schizophrenia patients from healthy controls. The AUC values and 95% confidence interval of CLIC3 was 0.69 (Figure 7H). Since the AUC value of CLIC3 was larger than 0.5, it was very likely that CLIC3 could be an indicator (biomarker) that is able to differentiate schizophrenia subjects from normal controls and could be a potential genetic marker for the clinical diagnosis of schizophrenia.

**Figure 8:**
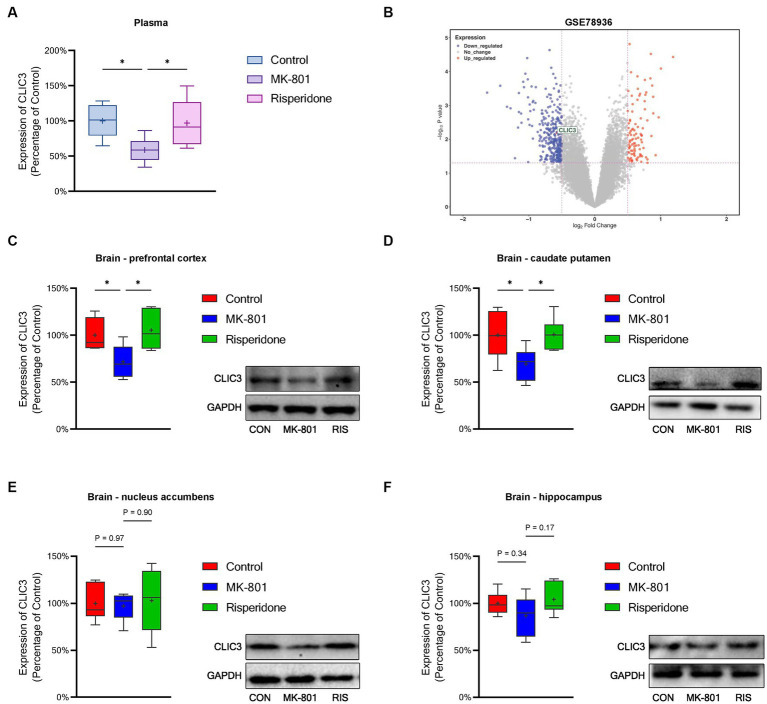
Protein expression of CLIC3 in the rat plasma and brains. **(A)** CLIC3 expression in the plasma. **(B)** CLIC3 mRNA expression in schizophrenia patients’ cortex. **(C)** CLIC3 expression in the prefrontal cortex (PFC). **(D)** CLIC3 expression in the caudate putamen (CPu). **(E)** CLIC3 expression in the nucleus accumbens (NAc). **(F)** CLIC3 expression in the hippocampus (HIP). *p < 0.05; +mean value.

To validate above bioinformatic results, we investigated CLIC3 expression in a schizophrenia animal model. First, the CLIC3 levels in the rat plasma were measured. The results showed that the levels of CLIC3 was significantly decreased in the model rats (ANOVA: *F*_2,15_ = 4.6, *p* < 0.05; *post hoc*: –41.6%, *p* < 0.05 vs. the control group) (Figure 8A). However, the risperidone administration elevated the CLIC3 expression to near normal levels. Additionally, we validated the expression of CLIC3 in the brain. First, we analyzed a post-mortem brain dataset (GSE78936) from the GEO database. This dataset recorded the mRNA expression of schizophrenia patients’ cortex (including orbitofrontal cortex, anterior cingulate cortex, and dorsolateral prefrontal cortex). The result showed that the CLIC3 expression was down-regulated in schizophrenia patients’ cortex (Figure 8B). Furthermore, we examined the protein expression of CLIC3 in rat schizophrenia rat brains. The expression of CLIC3 was presented in all four schizophrenia-related brain regions. Specifically, the repeated MK-801 administration significantly reduced the protein levels of CLIC3 in PFC (ANOVA: *F*_2,15_ = 5.84, *p* < 0.05; *post hoc*: –28.4%, *p* < 0.05 vs. the control group) and CPu of the model rats (ANOVA: *F*_2,15_ = 4.56, *p* < 0.05; *post hoc*: –30.8%, *p* < 0.05 vs. the control group) (Figures 8C,D). On the other hand, in the NAc and HIP, the expression of CLIC3 was not significantly altered by the MK-801 administration (both *p* > 0.05) (Figures 8E,F). It should also be noted that the antipsychotic drug - risperidone also eliminated the inhibitory effects of MK-801 on CLIC3 expression in both PFC and CPu (both *p <* 0.05 vs. the MK-801 group) (Figures 8A,B), indicating that CLIC3 might be a potential therapeutic target of schizophrenia.

## 4. Discussion

Schizophrenia is a group of extremely severe neurodevelopmental disorders, resulting in substantial economic and social burdens (McCutcheon et al., 2020). It has been accepted that early and accurate identification and diagnosis are critical for the clinical treatment of schizophrenia. Due to the poor understanding on the molecular basis of schizophrenia, exploration and identification of peripheral genetic markers in blood is imperative, which will greatly benefit the clinical diagnosis and treatment of schizophrenia. The present study analyzed four datasets of schizophrenia peripheral blood gene expression (GSE18312, GSE27383, GSE165604, and GSE38484). By using the WGCNA and DEG analyses and the KEGG and GO enrichment analyses, 317 DEGs were identified and also found to be related with immune processes. Subsequently, an immune infiltration analysis was performed using the CIBERSORT algorithm and revealed that 4 types of immune cells (eosinophils, M0 macrophages, resting mast cells, and gamma delta T cells) dramatically differed between the schizophrenia subjects and healthy controls. Moreover, three machine-learning algorithms including Random Forest, LASSO, and SVM-RFE were employed to further screen hub gene(s) from these 317 genes. Finally, 1 immune-related hub gene of schizophrenia - CLIC3 was identified, followed by the protein-level validations of CLIC3 in the brain of schizophrenia model rats. To our best knowledge, the present study is the first study that revealed the relationship between CLIC3 and schizophrenia.

In the present study, we first identified 317 differentially-expressed key genes associated with schizophrenia using the WGCNA and DEG analyses. The subsequent KEGG and GO enrichment analyses pointed out that these 317 key genes were mainly related to immune processes, such as, toll-like receptor signaling pathway, oxidative phosphorylation, apoptosis, leukocyte activation involved in immune response, cell activation involved in immune response, neutrophil activation involved in immune response, neutrophil mediated immunity. Since the samples examined in this study were blood lymphocytes or PBMC, these enrichment results were consistent with our speculation. It has been well-documented that immunity and inflammation are closely associated with the pathophysiology of schizophrenia (Khandaker et al., 2015). These findings confirmed that abnormal alteration in the immune system is strongly associated with schizophrenia and also indicated that the peripheral immune system is very likely to be a diagnostic indicator and therapeutic target for schizophrenia. Therefore, further research focusing on the peripheral immune system is going to be of great importance in the diagnosis and therapeutics of schizophrenia. However, it is also worth noting that the other molecular pathway revealed by the enrichment analyses such as ‘Sphingolipid signaling pathway,’ ‘Mitophagy,’ ‘Oxidative phosphorylation,’ ‘Apoptosis,’ and ‘COVID-19’ cannot be neglected and in-depth investigations on these pathways in schizophrenia are also required in the future.

CIBERSORT is a de-convolution machine learning algorithm used to estimate the proportion of 22 types of immune cells on the basis of gene expression. Since the 317 schizophrenia-associated key genes were related to various immune processes, the CIBERSORT algorithm were employed to identify related immune cell types. The data revealed that among the 22 immune cell types, 4 types of immune cells, including eosinophils, gamma delta T cell, M0 macrophages, and resting mast cells, were revealed to be significantly different in schizophrenia. Under inflammatory conditions, eosinophils were involved in immune regulation in neurons mainly through migrating to inflammatory sites and acting with various cytokines (Rothenberg and Hogan, 2006; Wechsler et al., 2021). In addition, gamma delta T cells were reported to be involved in the pathogenesis of a variety of neurological diseases, including multiple sclerosis, Parkinson’s disease (PD), Alzheimer’s disease (AD), cerebrovascular disease, epilepsy, and Rasmussen encephalitis (Fiszer, 1995; Al Nimer et al., 2018; Xu et al., 2018). Macrophages play a major role in innate immunity in the brain (Monji et al., 2013) and macrophage CD163 mRNA levels were found to be increased in schizophrenia patients, particularly in the high inflammation schizophrenia subgroup (Zhu et al., 2022). Lastly, mast cells were found in various brain regions, interacting with neurons, glia, blood vessels, and other hematopoietic cells (Dimitriadou et al., 1990; Manning et al., 1994; Silver and Curley, 2013). Up-regulation of mast cells induced by inflammation can lead to cognitive, socio-behavioral abnormalities and anxiety-like behavior (Nautiyal et al., 2008; Skaper et al., 2014; Breach et al., 2021). These findings suggest that these types of immune cells are very likely to be associated with the pathogenesis of schizophrneia and could be used as diagnostic biomarkers or therapeutic targets of schizophrenia in the future.

Although 317 schizophrenia-associated key genes were identified in the first section of this study, it is unfortunately almost impossible to use all of them to diagnosis schizophrenia or develop new drugs. It is necessary to investigate their importance or correlations with the pathogenesis of schizophrenia and then screen out one or several most important gene(s) for schizophrenia. Machine learning techniques have emerged from Artificial Intelligence (AI), which primarily sought to determine pattern recognition. Applications of machine learning in translational medicine include the development of novel drugs and treatments, diagnostic development, surgical planning, outcome prediction, and intraoperative assistance. At present, machine learning has become one of the most important methods for identifying critical genes, which can facilitate the identification of therapeutic targets and/or diagnostic biomarkers; at the same time, machine learning is considered as one of the major supplementary approaches to reduce the resources required for necessity measurement (Aromolaran et al., 2021). In the field of psychiatry, machine learning has been widely used for modeling and therapeutic discovery of various neuropsychiatric diseases, inducing Alzheimer’s disease, schizophrenia, Parkinson’s disease, depression disorders, *etc* (Tai et al., 2019). In the present study, three machine-learning algorithms were adopted to screen hub gene(s), including Random Forest, LASSO, and SVM-RFE. These three machine-learning algorithms are able to screen out and rank the genes that are very likely to be associated with schizophrenia based on the current data. Since these three machine-learning algorithms have their own intrinsic advantages and shortcomings, using only one algorithm would bring unpredictably biased screening results. Therefore, to avoid such biases, the shared gene(s) of the results of these three machine-learning algorithms were considered as hub gene(s) in the current study. As the results showed, only one gene was screened out by the three machine-learning algorithms, namely CLIC3.

CLIC3 has been found to be expressed in human placenta and fetal membranes (Money et al., 2007), osteoblasts (Brum et al., 2017), and various cancer cells (Patel et al., 2019; Chen et al., 2020; Kawai et al., 2020). Its rich expression promotes immune evasion in cancer cells (Vlachostergios et al., 2022). Addtionally, it was reported that in hepatitis B virus, CLIC3 promotes classical macrophage activation via the NF-κB pathway (Liang et al., 2022). Unfortunately, the exact role of CLIC3 in schizophrenia is not clear based on existing literature. Therefore, we established a schizophrenia animal model and examined the protein expression of CLIC3 in the plasma and brain. The results demonstrated the CLIC3 expression was decreased in the plasma and the PFC and CPu of the brains. These findings indicate that altered CLIC3 expression is very likely to be associated with schizophrenia and could be a potential biomarker of schizophrneia. However, in-depth investigations, especially the examination of CLIC3 expression in schizophrenia patients, are still required to further verify the role of CLIC3 in schizophrenia. Futhermore, since schizophrenic patients always experience a long history of various medications, it is possible that the alterations in the CLIC3 protein expression may not be observed in patients due to feedback regulation of the body. Therefore, CLIC3 expression in clinical samples such as post-mortem brain, peripheral blood, or cerebrospinal fluid, is required to be investigated in the future. On the other hand, employing a long-term schizophrenia animal model might also be an effective approach to examine the changes of CLIC3 in schizophrenia and validate our current findings.

In the present study, we found that risperidone was able to reverse the altered expression of CLIC3 in the plasma and brains of the schizophrenia model rats, suggesting that regulating CLIC3 expression might be a potential approach to treating schizophrenia and CLIC3 is a possible therapeutic target of risperidone. Nevertheless, it should be noted that although risperidone is a common-used antipsychtic drug, there are many other antipsychotic drugs in the market, such as haloperidol, aripiprazole, brexpiprazole, etc. These drugs possess different pharmacological mechanisms and are suitable for different clinical manifestations. Therefore, the effects of other antipsychotic drugs on CLIC3 are also required to be examined in the future. In addition, besides schizophrenia, risperidone is also used to treat symptoms of bipolar disorder and symptoms of irritability in autistic children (Chopko and Lindsley, 2018). Thus, whether CLIC3 is also a potential diagnostic biomarker and therapeutic target in bipolar disorder and autism is also a topic worth further exploring.

It is also worth noting that the current study used MK-801 to establish a NMDAR hyperfunction animal model, thus whether abnormal CLIC3 expression is associated with NMDAR dysfunction requires to be verified in future studies. Moreover, risperidone was widely reported to have positive effects on immune dysfunction (Richtand et al., 2011; Casquero-Veiga et al., 2019; Lokmer et al., 2023). Therefore, whether risperidone regulates the immune system through modulating CLIC3 expression is worth being explored in the future. It should be also noted that in the NAc and HIP, the CLIC3 expression was not significantly modulated, indicating that the alterations of CLIC3 expression are probably dependent on brain regions. Nevertheless, the exact relationship between CLIC3 expression and schizophrenia-related brain regions cannot be elucidated by the present data.

In conclusion, the current study identified 1 immune-related hub gene - CLIC3 and 4 peripheral immune cells (eosinophils, M0 macrophages, resting mast cells, and gamma delta T cells), which might be closely related to the pathogenesis of schizophrenia. In particular, CLIC3 has potential to be a promising biomarker or therapeutic target of schizophrenia. These findings together provide new insights for the diagnosis and therapeutics of schizophrenia.

## Data availability statement

The datasets presented in this study can be found in online repositories. The names of the repository/repositories and accession number(s) can be found in the article/Supplementary material.

## Ethics statement

Ethical approval was not required for the study involving humans in accordance with the local legislation and institutional requirements. Written informed consent to participate in this study was not required from the participants or the participants’ legal guardians/next of kin in accordance with the national legislation and the institutional requirements. The animal study was approved by Animal Ethics Committee of Yangzhou University Medical College. The study was conducted in accordance with the local legislation and institutional requirements.

## Author contributions

XZ: Investigation, Methodology, Writing – original draft, Writing – review & editing. C-lW: Writing – original draft, Conceptualization, Data curation, Formal analysis, Funding acquisition, Investigation, Methodology, Project administration, Writing – review & editing. J-fY: Writing – review & editing, Data curation, Formal analysis, Investigation, Methodology. JW: Writing – review & editing, Data curation, Formal analysis, Investigation, Methodology. BH: Writing – review & editing, Data curation, Formal analysis, Investigation, Methodology. YL: Writing – review & editing, Data curation, Formal analysis, Investigation, Methodology. XT: Conceptualization, Methodology, Writing – review & editing, Writing – original draft. BP: Writing – review & editing, Conceptualization, Data curation, Formal analysis, Funding acquisition, Investigation, Methodology, Project administration, Writing – original draft.

## Funding

The author(s) declare financial support was received for the research, authorship, and/or publication of this article. This work was supported by a Postgraduate Research & Practice Innovation Program of Jiangsu Province (SJCX22_1830), a Social Development Program of Hanjiang District of Yangzhou (2022), an Open Project of the Jiangsu Key Laboratory of Integrated Traditional Chinese and Western Medicine for Prevention and Treatment of Senile Diseases of Yangzhou University Medical College (202226), a Research Project of Jiangsu Commission of Health (M2020031), and an Elderly Health Research Project of Jiangsu Commission of Health (LR2022015 and LKZ2023020). The funding organizations did not play a role in study design and conduct, data interpretation, or paper writing.

## Conflict of interest

The authors declare that the research was conducted in the absence of any commercial or financial relationships that could be construed as a potential conflict of interest.

## Publisher’s note

All claims expressed in this article are solely those of the authors and do not necessarily represent those of their affiliated organizations, or those of the publisher, the editors and the reviewers. Any product that may be evaluated in this article, or claim that may be made by its manufacturer, is not guaranteed or endorsed by the publisher.

## Footnotes

^1^ https://www.ncbi.nlm.nih.gov/geo/

^2^ http://sangerbox.com/

^3^ https://cibersortx.stanford.edu/

^4^ https://pathcards.genecards.org/

^5^ https://www.immport.org/

^6^ https://CRAN.R-project.org/package=e1071
