# Supplementary material for: Identification of immune-related biomarkers in peripheral blood of schizophrenia using bioinformatic methods and machine learning algorithms
Source: Front Cell Neurosci. 2023 Sep 28;17:1256184. doi: 10.3389/fncel.2023.1256184 (PMC10568181; doi:10.3389/fncel.2023.1256184)
Supplement: Supplementary file 1 [file Data_Sheet_1.PDF]

Figure S1
(A)

PFC

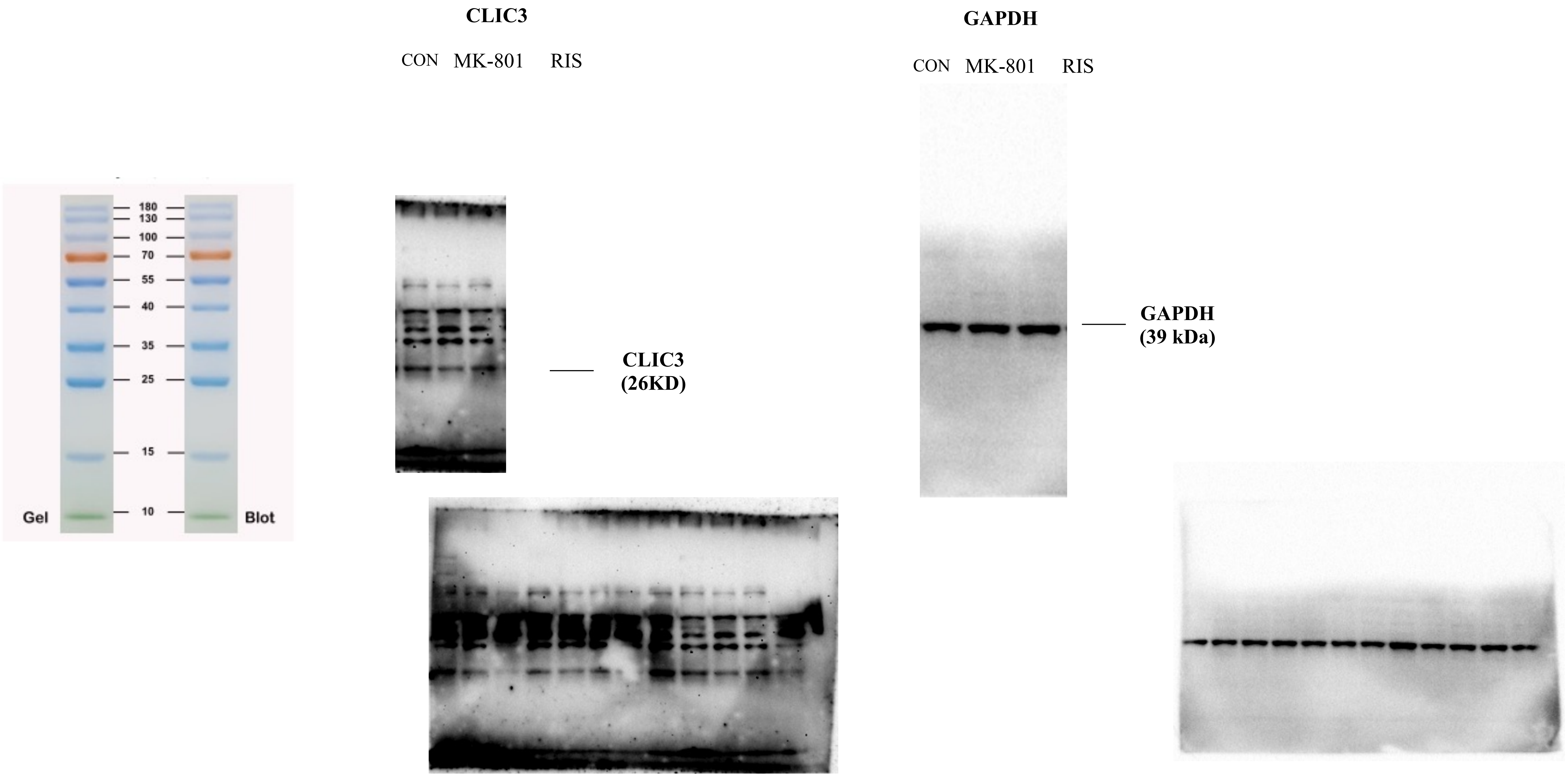

Figure S1
(B)

CPu

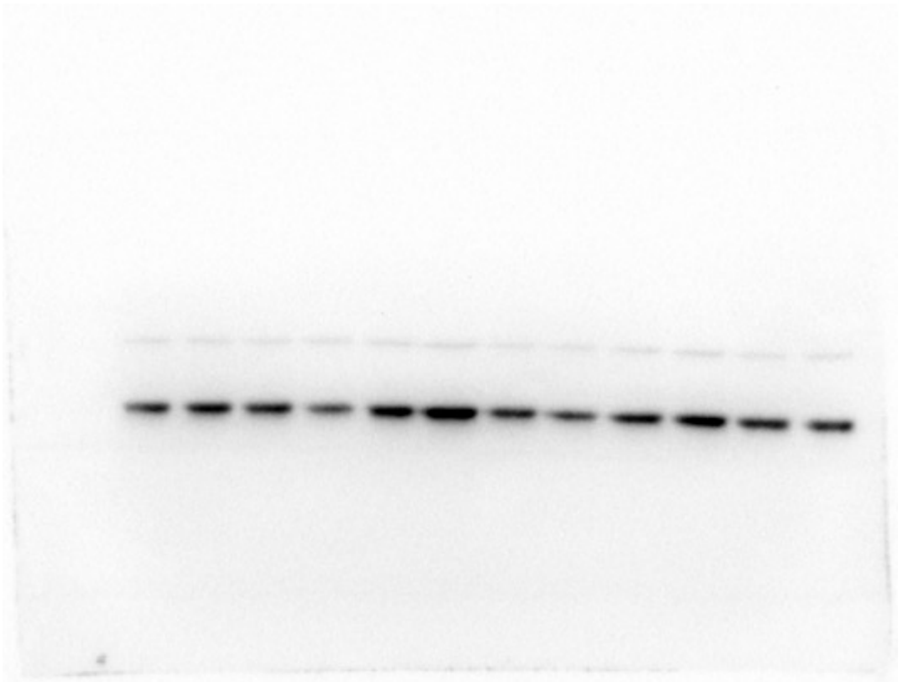

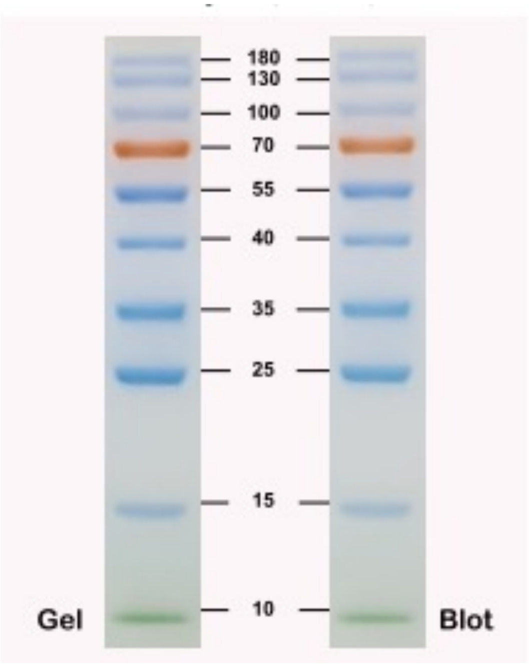

CLIC3

CON MK-801 RIS

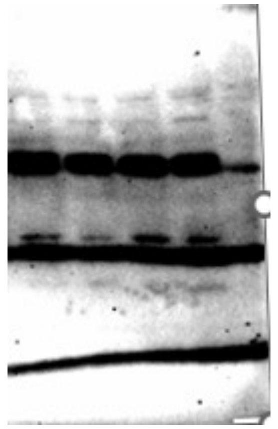

CLIC3 (26KD)

GAPDH

CON MK-801 RIS

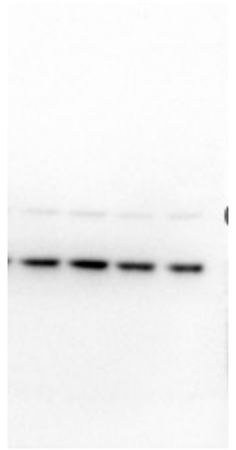

GAPDH (39 kDa)

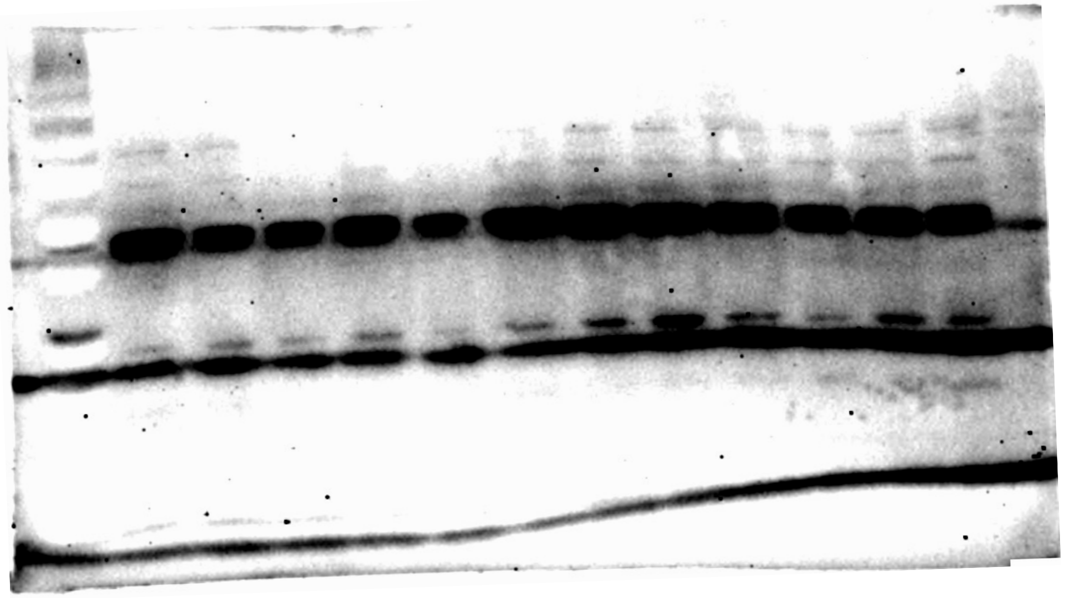

Figure S1
(C)

NAc

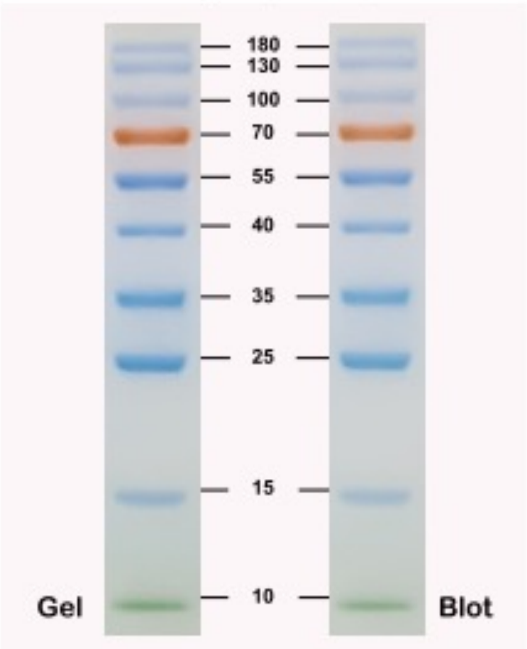

CLIC3

CON MK-801 RIS

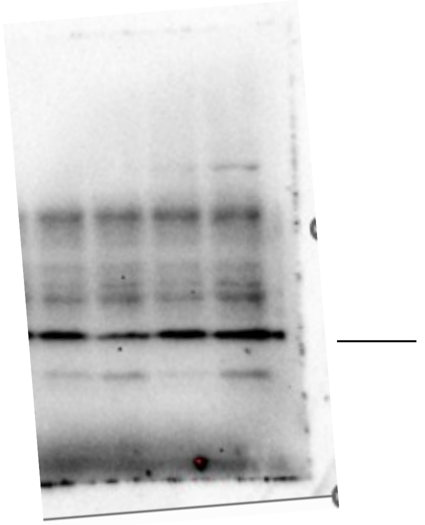

CLIC3 (26KD)

GAPDH

CON MK-801 RIS

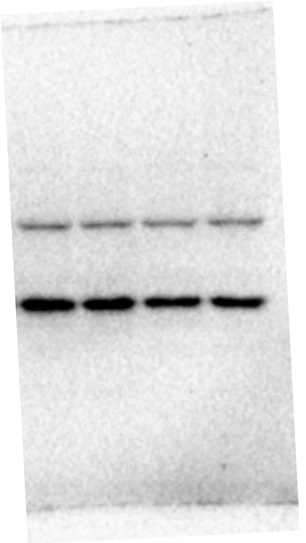

GAPDH (39 kDa)

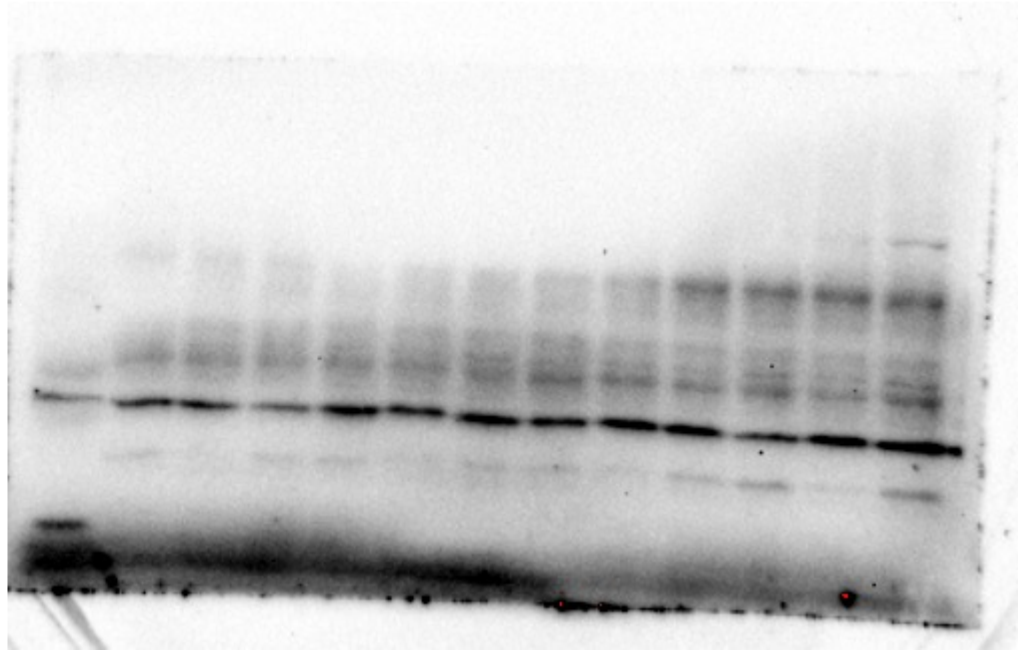

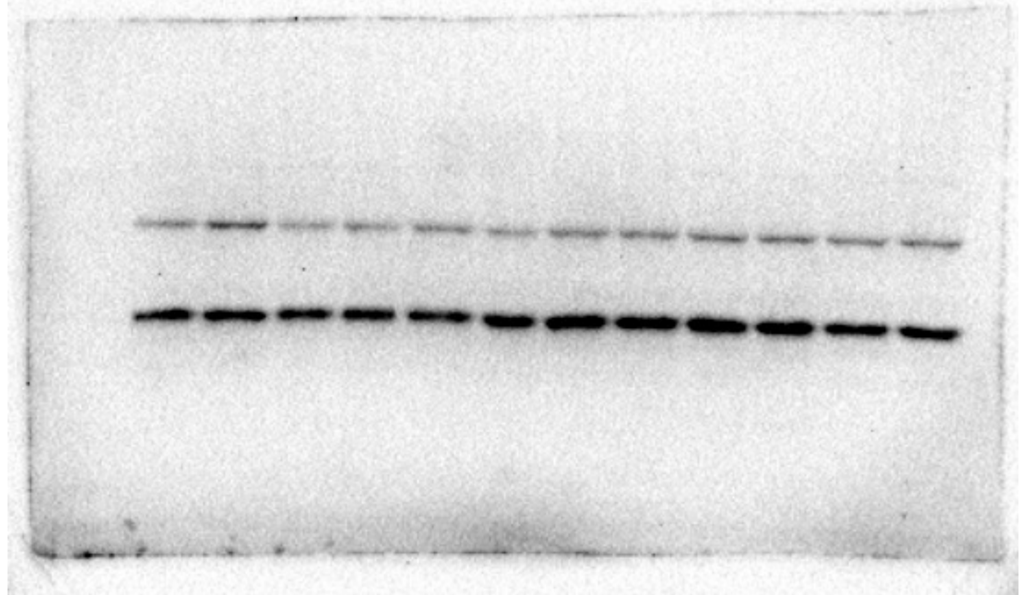

Figure S1
(D)

HIP

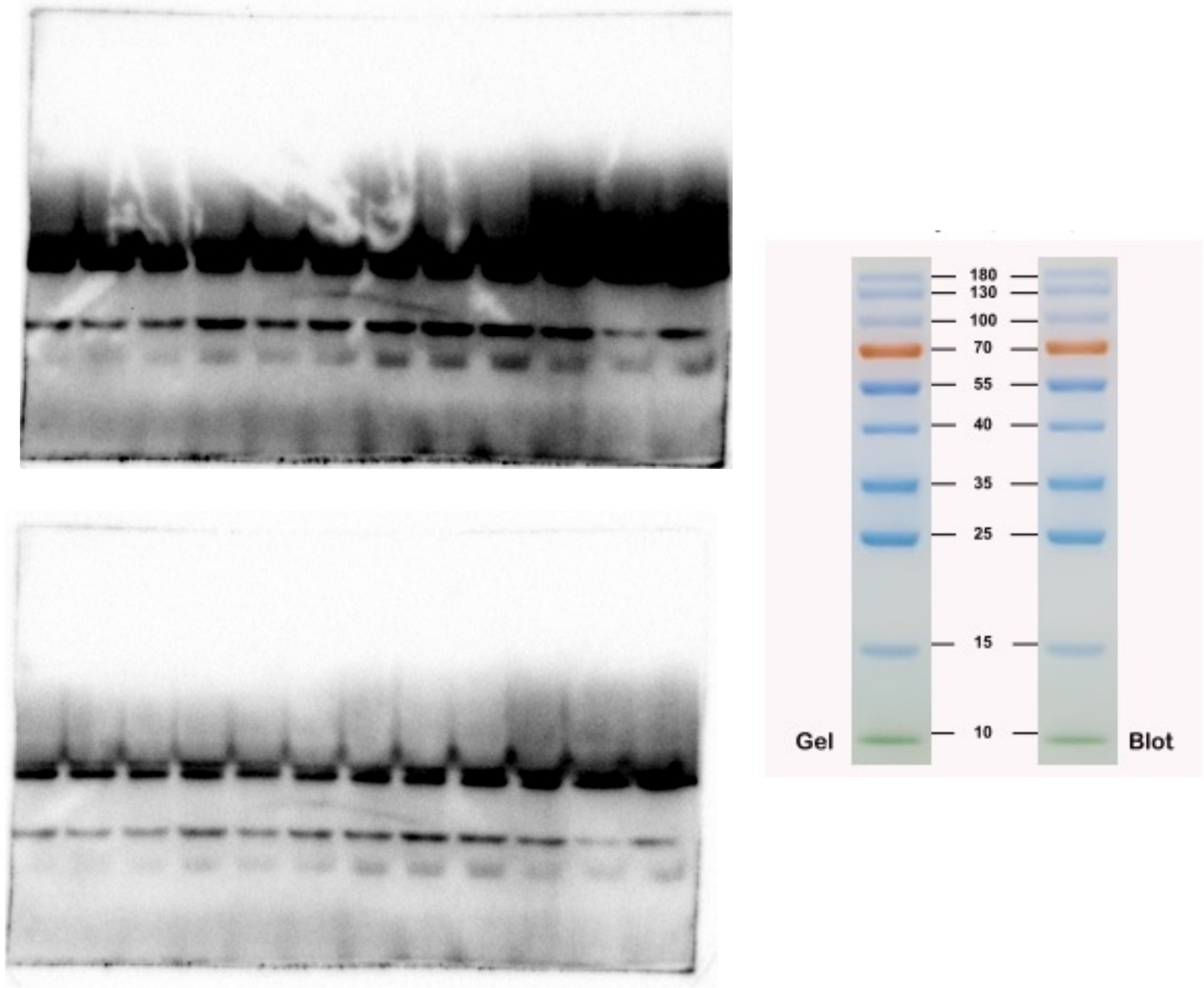

CLIC3

CON MK-801 RIS

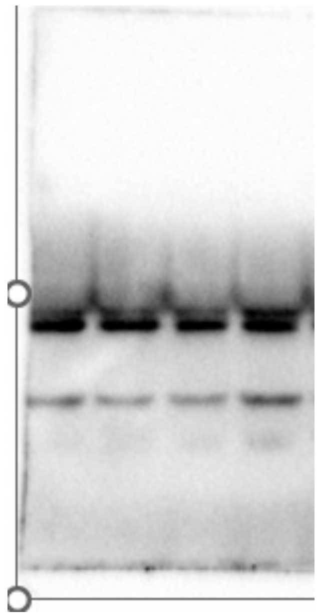

CLIC3
(26KD)

GAPDH

CON MK-801 RIS

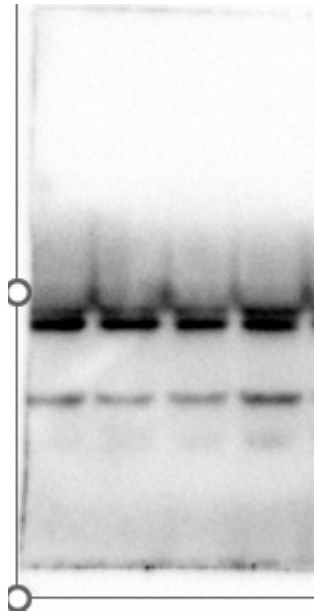

GAPDH
(39 kDa)
